# Transfer learning of pre-treatment quantitative ultrasound multi-parametric images for the prediction of breast cancer response to neoadjuvant chemotherapy

**DOI:** 10.1038/s41598-024-52858-y

**Published:** 2024-01-29

**Authors:** Omar Falou, Lakshmanan Sannachi, Maeashah Haque, Gregory J. Czarnota, Michael C. Kolios

**Affiliations:** 1 Department of Physics, Toronto Metropolitan University, Toronto, ON Canada; 2 Institute for Biomedical Engineering, Science and Technology (iBEST), Keenan Research Centre for Biomedical Science, St. Michael’s Hospital, Toronto, ON Canada; 3 Department of Radiation Oncology, Sunnybrook Health Sciences Centre, Toronto, ON Canada; 4 Department of Radiation Oncology, University of Toronto, Toronto, ON Canada; 5 Physical Sciences, Sunnybrook Research Institute, Toronto, ON Canada; 6 Department of Medical Biophysics, University of Toronto, Toronto, ON Canada

**Keywords:** Cancer imaging, Predictive markers

## Abstract

Locally advanced breast cancer (LABC) is a severe type of cancer with a poor prognosis, despite advancements in therapy. As the disease is often inoperable, current guidelines suggest upfront aggressive neoadjuvant chemotherapy (NAC). Complete pathological response to chemotherapy is linked to improved survival, but conventional clinical assessments like physical exams, mammography, and imaging are limited in detecting early response. Early detection of tissue response can improve complete pathological response and patient survival while reducing exposure to ineffective and potentially harmful treatments. A rapid, cost-effective modality without the need for exogenous contrast agents would be valuable for evaluating neoadjuvant therapy response. Conventional ultrasound provides information about tissue echogenicity, but image comparisons are difficult due to instrument-dependent settings and imaging parameters. Quantitative ultrasound (QUS) overcomes this by using normalized power spectra to calculate quantitative metrics. This study used a novel transfer learning-based approach to predict LABC response to neoadjuvant chemotherapy using QUS imaging at pre-treatment. Using data from 174 patients, QUS parametric images of breast tumors with margins were generated. The ground truth response to therapy for each patient was based on standard clinical and pathological criteria. The Residual Network (ResNet) deep learning architecture was used to extract features from the parametric QUS maps. This was followed by SelectKBest and Synthetic Minority Oversampling (SMOTE) techniques for feature selection and data balancing, respectively. The Support Vector Machine (SVM) algorithm was employed to classify patients into two distinct categories: nonresponders (NR) and responders (RR). Evaluation results on an unseen test set demonstrate that the transfer learning-based approach using spectral slope parametric maps had the best performance in the identification of nonresponders with precision, recall, F1-score, and balanced accuracy of 100, 71, 83, and 86%, respectively. The transfer learning-based approach has many advantages over conventional deep learning methods since it reduces the need for large image datasets for training and shortens the training time. The results of this study demonstrate the potential of transfer learning in predicting LABC response to neoadjuvant chemotherapy before the start of treatment using quantitative ultrasound imaging. Prediction of NAC response before treatment can aid clinicians in customizing ineffectual treatment regimens for individual patients.

## Introduction

Locally advanced breast cancer is a severe type of cancer with a poor prognosis, despite advancements in therapy. In the US alone, the American Cancer Society reports 287,850 new cases of invasive breast cancer, 51,400 new cases of carcinoma in situ, and 43,250 deaths each year^1^. LABC affects mainly young women and results in a significant loss of life and a burden for families and society. As the disease is often inoperable, current guidelines suggest upfront aggressive neoadjuvant chemotherapy^2–5^. These patients have a 2- to 5-year survival rate of 30–60%, with many experiencing local recurrences in addition to metastatic progression^3^. This implies that 40–70% of chemotherapies administered to such patients are ultimately ineffective in terms of response rates and long-term survival. Complete pathological response to chemotherapy is linked to improved survival, but conventional clinical assessments like physical exams, mammography, and imaging are limited in detecting early response. Early detection of tissue response can improve complete pathological response and patient survival while reducing exposure to ineffective and potentially harmful treatments.

MRI and PET scans have been used as tools for response prediction, but they are expensive and require contrast agents and long scan times^6^. A rapid, cost-effective modality without the need for contrast agents would be valuable for evaluating neoadjuvant therapy response. Conventional ultrasound provides information about tissue echogenicity, but image comparisons are difficult due to different hardware configurations and instrument settings. Quantitative ultrasound overcomes this by using normalized power spectra to calculate quantitative metrics, including Average Scatterer Diameter (ASD), Average Acoustic Concentration (AAC), Midband Fit (MBF), Spectral Slope (SS), and Spectral Intercept (SI), which can characterize tumors and assist in treatment evaluation. These QUS spectral characteristics have been shown to correlate with patients’ response to NAC both prior to and after chemotherapy initiation^7–10^. In addition, QUS spectroscopy has been used to monitor therapy response by comparing the QUS parameters acquired at week 0 (baseline) to those acquired at weeks 1, 4, and 8 after the initiation of NAC^10,11^.

Deep learning is a subfield of artificial intelligence that uses algorithms inspired by the structure and function of the brain to analyze images. These algorithms, known as artificial neural networks, can remove the process of extracting handcrafted features from images. Deep learning has been used in image analysis to perform object detection, image segmentation, and classification tasks. In a recent study, Taleghamar et al.^7^ investigated a deep-learning approach to predict LABC to NAC using quantitative ultrasound imaging from 181 patients at pre-treatment. Different convolutional neural network (CNN) architectures were investigated for feature extraction, including Residual Attention Networks (RAN) and ResNets. In a set of experiments, the feature maps were extracted from the tumor core and the core and its margin. After averaging the features from various tumor cross-sections, a fully connected network was used for response prediction. The ground truth response to NAC was identified for each patient after the surgery using the standard clinical and pathological criteria. Their results demonstrated that their developed model with the RAN architecture has an accuracy of 88% on the test set. However, their model was trained on a limited number of images, which may have contributed to the decrease in the model accuracy when applied to new, unseen data. Moreover, their training dataset was imbalanced (43 nonresponders vs. 138 responders) and hence biased towards the responders’ class. A balanced and diverse dataset is crucial for deep learning models to perform well. In this work, we propose to use the transfer learning approach to predict treatment response. Transfer learning is a machine learning technique where a model trained on one task is fine-tuned on a second task using a smaller number of training examples compared to training a model from scratch on the second task^12^. This enables the model to leverage its knowledge of the first task to learn the second task quickly and helps overcome the problem of insufficient training data for the second task.

## Results

### Patient, tumor, and treatment characteristics

The pathological and clinical characteristics of locally advanced breast cancer patients undergoing neoadjuvant chemotherapy can be summarized in Table 1. The study patients had an average age of 51 years. The patients exhibited an initial average tumor size of 5.21 cm, which decreased to a residual average tumor size of 2.70 cm following the treatment. Of all the 174 patients, 137 were classified as responders, and the remaining 37 were designated as nonresponders. Histology indicates that 91% of patients were diagnosed with Invasive Ductal Carcinoma, 3% with Invasive Lobular Carcinoma, and the remaining 6% with Invasive Mixed Carcinoma. In terms of tumor grade, 6% of patients were classified as Grade I, 37% had Grade II tumors, and 48% had Grade III tumors, with the remnants of 9% not reported. As a result of systemic therapy, 42% of patients were subjected to AC-T (Adriamycin, Cyclophosphamide, and Paclitaxel), 21% with FEC-D (5-Fluorouracil, Epirubicin, Cyclophosphamide, and Docetaxel), and 37% with other chemotherapy regimens. Tumor distinction was performed utilizing molecular subtypes, including ERBB2 + (ER −, PR −, HER2 +), triple-negative (ER −, PR −, HER2 −), Luminal-A (ER + and/or PR +, HER2 −), and Luminal-B (ER + and/or PR + , HER2 +). Within the responder cohort, the distribution of molecular subtypes was as follows: 15% were classified as ERBB2 +, 27% as triple negative, 32% as Luminal-A, and 26% as Luminal-B. Among the nonresponder cohort, the breakdown of molecular subtypes was as follows: 0% were categorized as ERBB2 +, 22% as triple negative, 65% as Luminal-A, and 14% as Luminal-B.

**Table 1:** Pathological and clinical characteristics of LABC patients receiving NAC.

	NR (N = 37)	RR (N = 137)	All (N = 174)
Age	53 ± 11	51 ± 12	51 ± 11
Menopause (Χ^2^ = 2.324, *p* = 0.313)			
Postmenopausal (%)	41	49	47
Premenopausal (%)	51	38	41
Perimenopausal (%)	8	13	12
Initial tumor size (cm)	5.23 ± 2.64	5.21 ± 2.84	5.21 ± 2.79
Histology (Χ^2^ = 5.545, *p* = 0.063)			
IDC (%)	81	93	91
ILC (%)	8	2	3
IMC (%)	11	4	6
Tumor grade (Χ^2^ = 4.840, *p* = 0.184)			
Grade I (%)	11	5	6
Grade II (%)	46	35	37
Grade III (%)	41	50	48
Not Reported (%)	3	10	9
Molecular subtype (Χ^2^ = 15.737, *p* = 0.001)			
ERBB2 + (%)	0	15	11
Triple Negative (%)	22	27	26
Luminal-A (%)	65	32	39
Luminal-B (%)	14	26	24
Treatment (Χ^2^ = 10.957, *p* = 0.533)			
ACT (%)	51	39	42
FECD (%)	24	20	21
Others (%)	24	41	37
Residual tumor size (cm)	6.26 ± 4.71	1.73 ± 2.24	2.70 ± 3.47

The statistical chi-square analysis completed with the response type (NR and RR) exhibited the following results: menopause (Χ^2^ = 2.324, *p* = 0.313), histology (Χ^2^ = 5.545, *p* = 0.063), tumor grade (Χ^2^ = 4.840, *p* = 0.184), molecular subtype (Χ^2^ = 15.737, *p* = 0.001) and treatment (Χ^2^ = 10.957, *p* = 0.533).

### Quantitative ultrasound parametric maps

**Figure 1:**
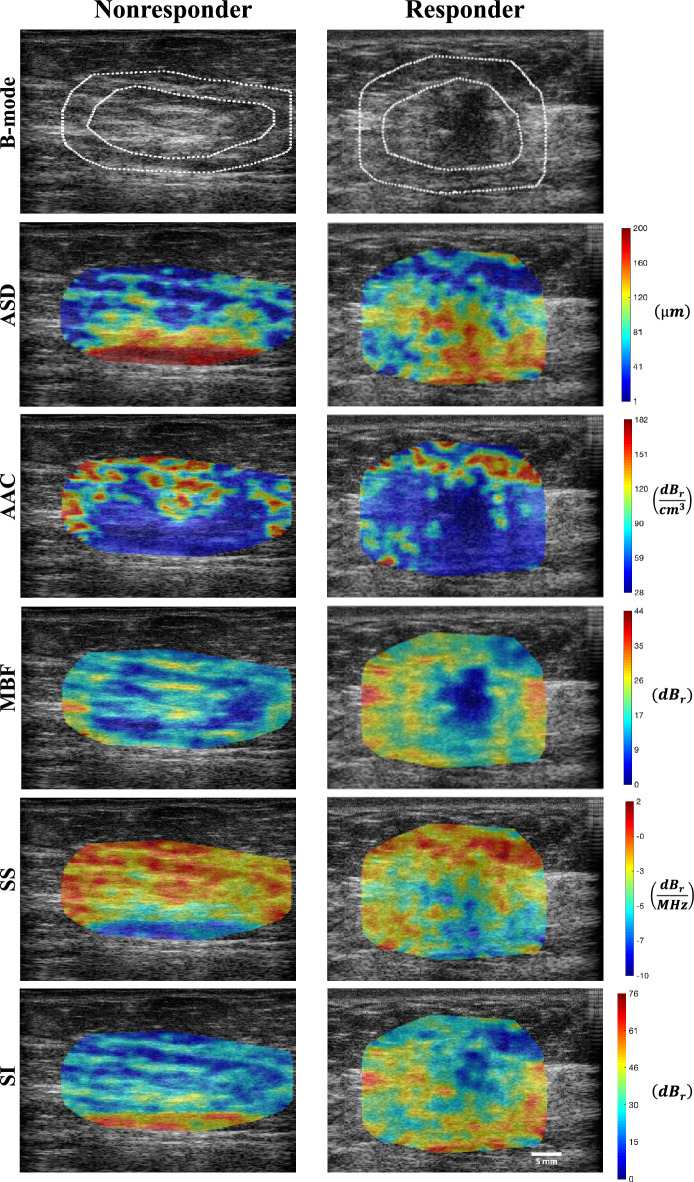
Representative B-mode, ASD, AAC, MBF, SS and SI parametric images with tumor core and margin regions of a nonresponder and a responder at pretreatment. The color bars indicate the values of the respective parameters across the tumor regions, each expressed through its corresponding unit. The white scale bar represents 5 mm.

Figure 1 shows QUS parametric maps of ASD, AAC, MBF, SS, and SI overlaid on the ultrasound B-mode images obtained from representative nonresponding and responding patients at pretreatment. A tumor in a B-mode image of a LABC patient’s breast can readily be identified as a hypo-intense mass surrounded by relatively hyper-intense fibroglandular tissue. Parametric maps of ASD, AAC, MBF, SS, and SI hold further information about the tumor, with each region (core and margin) containing a unique textural pattern.

### Classification performance

**Figure 2:**
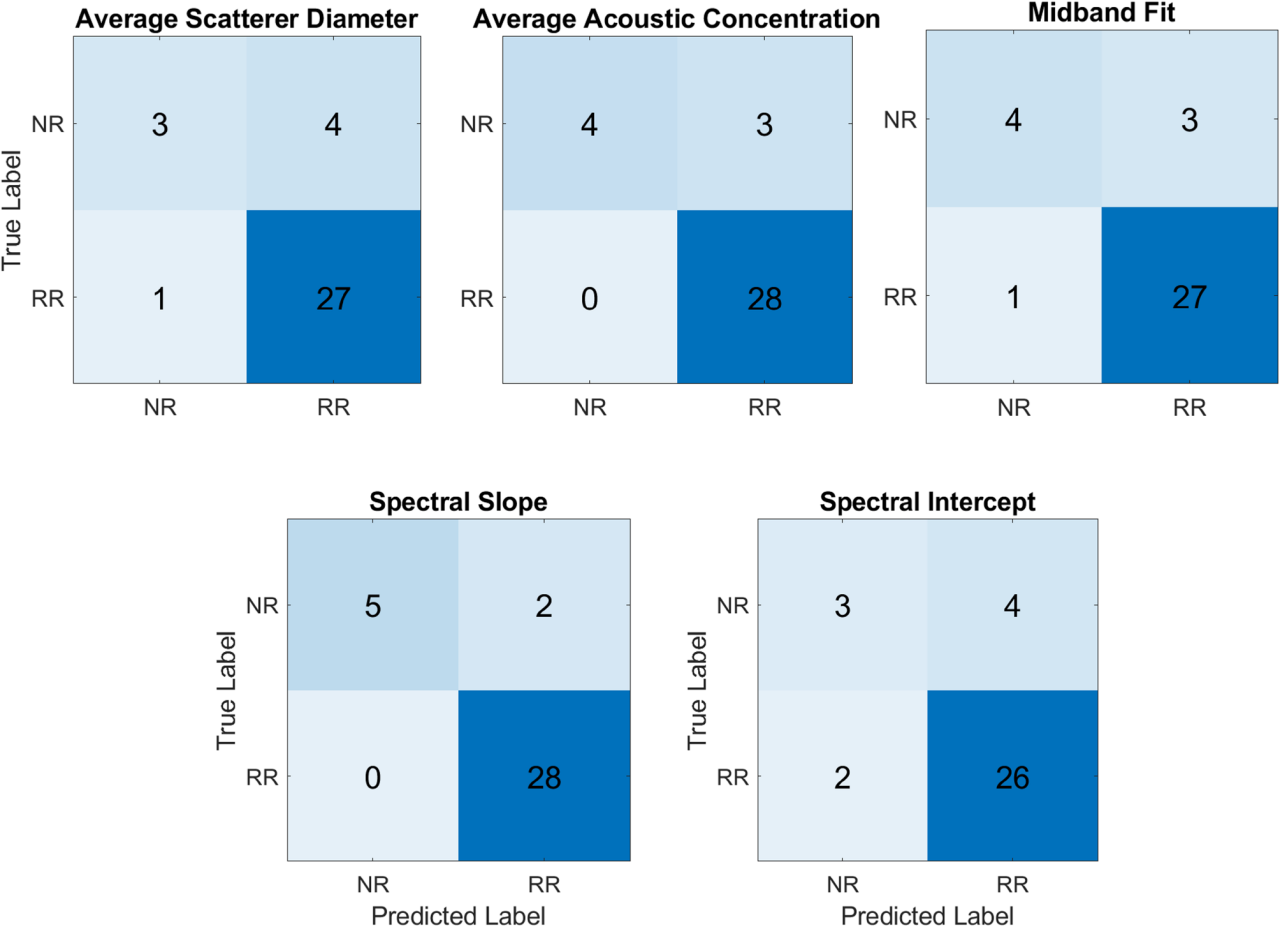
Confusion matrices of response prediction on the unseen dataset.

Figure 2 shows the confusion matrices of response prediction on the unseen dataset. For the spectral slope, the developed model is capable of perfectly predicting all responders. Out of seven nonresponders, five are accurately predicted.

Table 2 shows the results of response prediction on the unseen dataset. Among all QUS parametric images, the model developed based on spectral slope parametric images performed the best in response prediction before treatment with, precision, recall, and F1-score of 100%, 71%, 83%, 93%, 100%, and 97% for the nonresponding and responding patients, respectively. The overall accuracy of the proposed model achieved an accuracy of 86% on the unseen dataset.

**Table 2:** Performance metrics of the proposed model based on the unseen dataset.

QUS	Patient	Precision (%)	Recall (%)	F1-score (%)	Balanced accuracy (%)
ASD	NR	75	43	55	70
	RR	87	96	92	
AAC	NR	100	57	73	79
	RR	90	100	95	
MBF	NR	80	57	67	77
	RR	90	96	93	
SS	NR	100	71	83	86
	RR	93	100	97	
SI	NR	60	43	50	68
	RR	87	93	90	

### Survival curves

**Figure 3:**
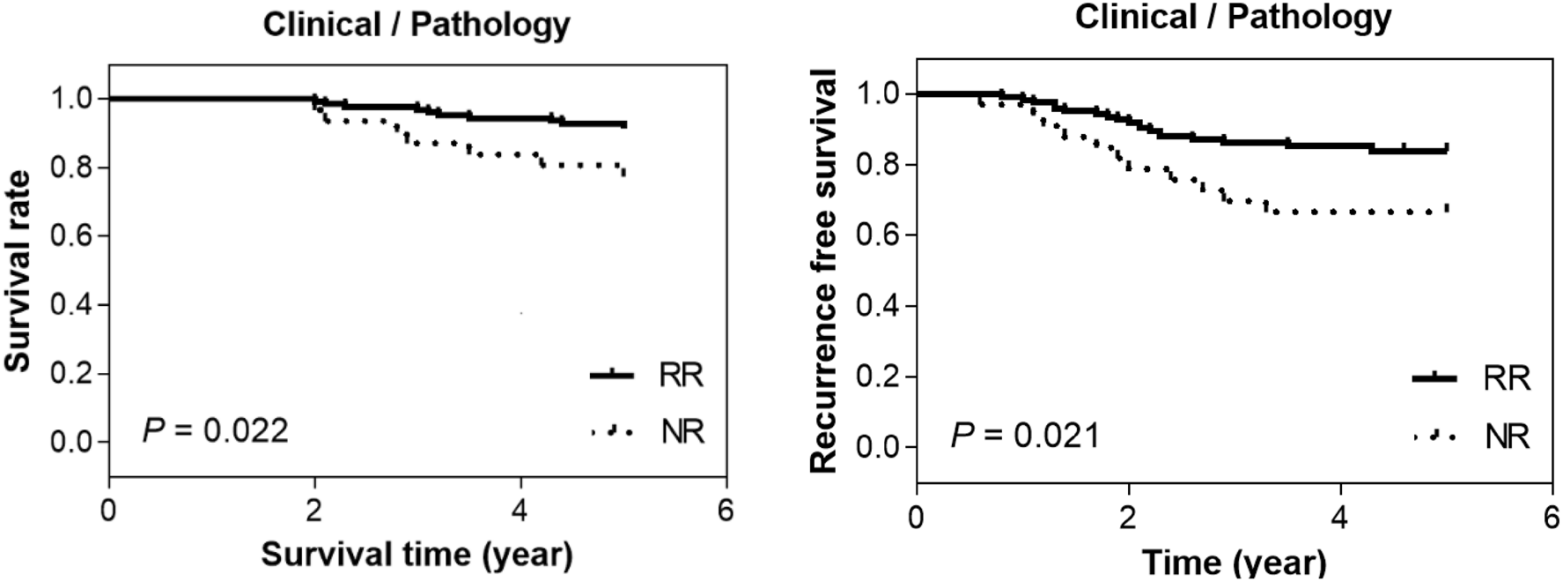
Overall survival and recurrence free survival curves for the two patient groups based on clinical and pathological criteria.

The overall survival and recurrence free survival curves for the two patient groups based on the clinical and pathological criteria are shown in Fig. 3. The five-year survival rates for responders and nonresponders are 96% and 88%, respectively. In terms of recurrence-free survival, responders exhibit a rate of 91%, while nonresponders show a lower rate of 82%. Differences between the two response groups are statistically significant ($p<0.05$).

### Discussion and conclusion

This study investigated a novel transfer learning-based approach for predicting LABC response to neoadjuvant chemotherapy using QUS imaging at pre-treatment. The ground truth response to therapy for each patient was based on standard clinical and pathological criteria. The ResNet50V2 deep learning architecture was used to extract features from the parametric maps. This was followed by SelectKBest and SMOTE techniques for feature selection and data balancing, respectively. The SVM algorithm was employed to categorize patients into two distinct groups: responders and nonresponders. The developed model was evaluated using four performance metrics on the unseen dataset. These include precision, recall, F1-score, and balanced accuracy. The results of the transfer learning-based approach on an unseen dataset demonstrated that spectral slope parametric maps performed the best in response prediction, with precision, recall, and F1-score of 100%, 71%, 83%, 93%, 100%, and 97% for the nonresponding and responding patients, respectively. The balanced accuracy of the proposed model is 86%.

It has been hypothesized that QUS-detected responses in tumors to cancer therapy are related to biological alterations in tumor microstructure and spatial heterogeneity. This led to several preclinical and clinical QUS studies that were conducted to detect tumor response to treatment early and during the course of treatment^7,13,14^. In these studies, QUS spectral parameters such as MBF, SS, and SI demonstrated a significant correlation with tumor response to treatment. These spectral parameters are associated with scatterer characteristics, such as scatterer size and scatterer acoustic concentration^15^. Similarly, ASD and AAC, which are estimated from the ultrasound backscatter coefficient by fitting a spherical Gaussian model to the measured backscatter coefficient, have been used to monitor tumor response in LABC patients undergoing NAC^15^. Moreover, textural features determined from QUS parametric maps, such as contrast, correlation, energy, and homogeneity have been shown to predict tumor treatment response in breast cancer patients^11,14^. In these studies, manually engineered features, such as the statistical and textural features of the QUS parametric maps, were extracted and utilized for response prediction using conventional machine learning techniques. While the manual extraction process of such features is simple, easier to understand and analyze, it has its own drawbacks. First, it frequently necessitates human intuition and domain expertise, which can be time-consuming. In addition, manually engineered features frequently require customization for particular datasets and may not generalize well. Convolutional Neural Networks, on the other hand, learn features directly from images, allowing them to acquire pertinent information for the specific task at hand, such as treatment response prediction. Moreover, CNNs learn hierarchical data representations. Through multiple levels of abstraction, they automatically discover and represent both low-level and high-level image characteristics. This ability to learn complex representations enables CNNs to capture intricate patterns and relationships in the images, which frequently results in higher accuracy.

**Figure 4:**
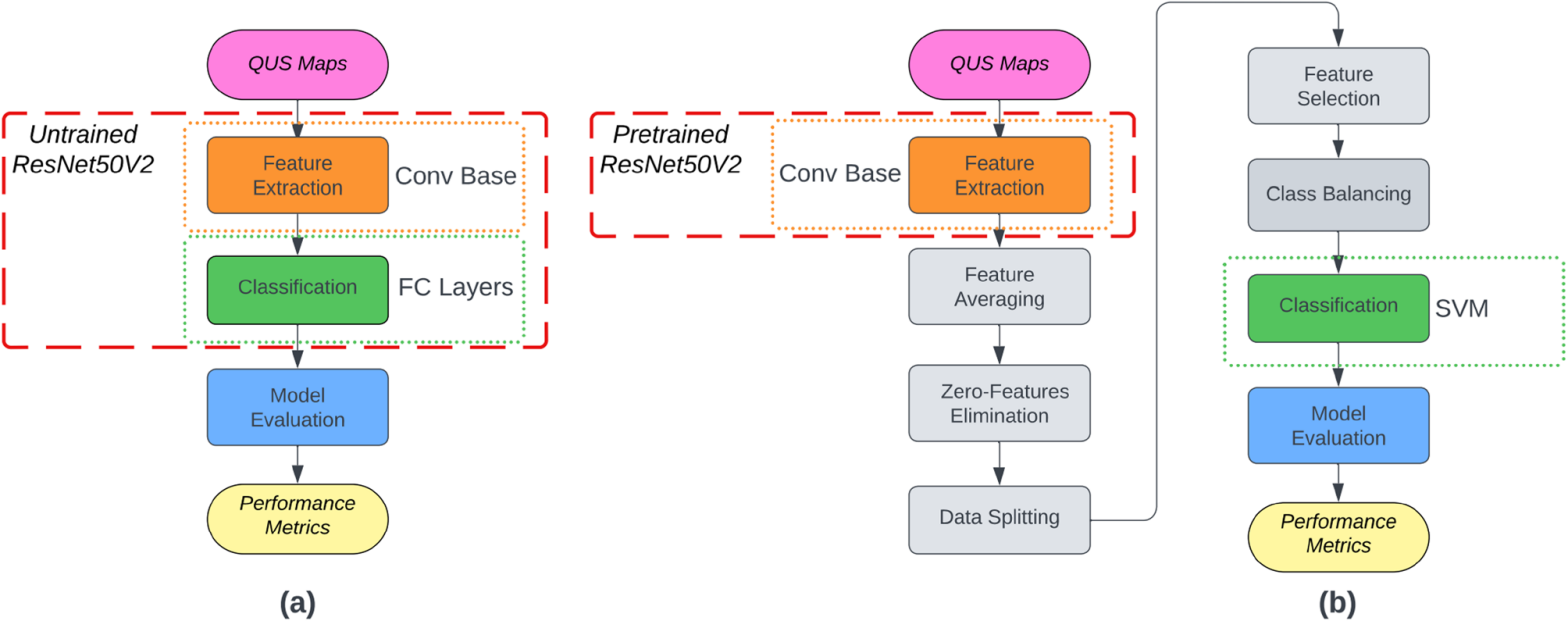
(**a**) Traditional deep learning image classification (**b**) Proposed approach using transfer learning.

In a recent study, Taleghamar et al.^7^ used a deep learning model to predict tumor response to treatment in patients with breast cancer using QUS before treatment. Different convolutional neural network architectures were used to extract features from QUS parametric maps. A fully connected network was used for treatment response prediction. Their developed models achieved an accuracy of 88% on an unseen dataset. However, their approach is computationally expensive and requires substantial amounts of labeled images for training which makes it unfavorable for the current problem. Transfer learning offers many advantages over traditional deep learning as it enables the use of models that have already been trained on vast datasets. By starting with pre-trained models, training time and resources can be significantly reduced. Moreover, it only requires a small, labeled dataset, as the pre-trained model has already learned generic features. In this work, the ResNet50’s lower layers were used to capture generic features from QUS maps, while the higher layers were replaced with SVM classifier to predict the treatment response of breast cancer patients (Fig. 4).

Among the various available architectures in computer vision applications, the ResNet was chosen for feature extraction since it employs skip connections that allow for much deeper architectures than conventional CNNs. Deeper networks can capture more intricate features and learn more abstract representations, resulting in enhanced performance on difficult tasks such as tumor response prediction. From the ResNet family of pre-trained models (such as ResNet50V2, ResNet101V2, and ResNet152V2), the ResNet50V2 was used since it has a small size (50 layers) and hence has better generalization capabilities and a lower risk of overfitting when compared to other pre-trained ResNet family models.

Precision (also called Positive Predictive Value or PPV) measures the proportion of correctly classified positive instances out of the total instances predicted as positive. It is a measure of the classifier's ability to avoid false positives. Recall (also called Sensitivity or True Positive Rate) measures the proportion of correctly classified positive instances out of the total actual positive instances. It is a measure of the classifier's ability to capture positive instances. The F1-score is the harmonic mean of precision and recall. It provides a balanced measurement that incorporates both metrics and is especially useful when there is an imbalance between positive and negative occurrences such as in this dataset. Balanced accuracy computes the mean of the per-class accuracies while accounting for the imbalance in class sizes. It provides an overall measurement of the efficacy of the classifier across all classes. Since we are specifically focused on identifying the nonresponders, a positive case indicates that the patient falls into the nonresponder category. The proposed model has a precision, a recall, an F1-score, and a Balanced Accuracy of 100%, 71%, 83%, and 86%, respectively using the SS parametric maps. This indicates that this approach has a 71% chance of predicting a nonresponding patient while avoiding incorrectly classifying responding patients as nonresponders. This is essential to ensure that the chemotherapeutic treatment regimen is administered to all potentially responding patients. When dealing with unbalanced data, the F1-score performance metric is frequently considered superior to accuracy. In this study, the responders’ class (137) outnumbers the nonresponders’ class (37), therefore it is possible to obtain a high level of accuracy by simply predicting the majority class (responders) for all instances. This can, however, result in an inability to correctly identify instances of the minority class (nonresponders). Incorrectly predicting the nonresponders, implies the administration of an unnecessary treatment which could lead to missed opportunities to enhance health outcomes.

Further analysis of larger datasets, stratified by various NAC regimens and possibly various molecular subtypes, may result in more accurate predictive models. The proposed model could also facilitate randomized clinical trials exploring NAC regimen modifications for patients with a low probability of responding to standard interventions. In conclusion, this investigation demonstrates the adaptability of transfer learning for predicting therapy response using quantitative imaging at pretreatment. The devised transfer learning model accurately predicts the response of patients with locally advanced breast cancer to NAC. Prediction of NAC response prior to treatment can aid clinicians in customizing ineffectual treatment regimens for individual patients. These encouraging results motivate further investigation of alternative transfer learning architectures and larger, multi-institutional patient cohorts to evaluate the robustness of these methodologies in clinical settings.

## Methods

### Study protocol

This investigation was conducted in accordance with the rules and regulations established by the Sunnybrook Health Sciences Centre’s institutional research ethics board and registered with ClinicalTrials.gov (NCT00437879). All experimental protocols were reviewed and approved by the Sunnybrook Research Institute research ethics board prior to commencing the study. All patients were enrolled with informed consent. The trial was open to 174 women between the ages of 27 and 83 who were diagnosed with LABC and scheduled for NAC and surgery.

All patients were subjected to a core needle biopsy prior to treatment as part of their standard of care to confirm a cancer diagnosis, histological subtype, and hormone receptor status to determine the tumor molecular subtype. In order to ascertain the initial tumor size prior to treatment, magnetic resonance images were acquired using a 1.0-T clinical MRI (GE Healthcare, Waukesha, WI) as part of the institutional standard of care for such patients. Ultrasound data was collected soon before patients began treatment. Mastectomy specimens were prepared onto a 5″ × 7″ whole-mount pathology slide and digitized using a confocal scanner (TISSUEscope™, Huron Technologies, Waterloo, ON) after surgery. A board-certified pathologist evaluated the samples and documented the findings in the patient's medical record.

Using a modified response grading system, patients were divided into responder and nonresponder groups based on the clinical/pathological tumor response determined at the conclusion of their treatment^11,14^. A response is defined as the disappearance of all target lesions, and any pathological lymph nodes must have a reduction in short axis to < 10 mm or at least a 30% decrease in diameter of target lesions or cellularity < 5% in the tumor bed (invasive disease) irrespective of size. This category incorporates both complete responders and partial responders. On the other hand, a non-response indicates less than a 30% reduction in tumor size and no significant alterations in tumor cellularity. This category incorporated stable disease and progressive disease.

### Ultrasound data acquisition and parametric maps generation

Ultrasound RF data was acquired before the start of the treatment using an RF-enabled Sonix RP system (Analogic Medical Corp., Vancouver, Canada) equipped with an L14-5/60 transducer. The transducer operated at the center frequency of 6 MHz with a − 6 dB bandwidth of 3–8 MHz. For each patient, ultrasound data was collected at four to seven image planes across the tumor, with approximately one centimeter between each image plane with the transducer focused towards the center of the tumor. The lateral and axial dimensions of the image were 6 cm and 4–6 cm, respectively. Ultrasound data was digitally collected with a sampling frequency of 40 MHz with a 16-bit resolution. On each ultrasound frame, a region of interest (ROI) was manually contoured corresponding to the tumor under the supervision of expert oncologists.

Standardization methods and the calculation of quantitative ultrasound parameters have been described in detail in previous work^16^ using a MATLAB-based (MathWorks, Natick, MA) custom software. In brief, the Fourier transform of the Hanning-gated RF data was obtained for each scan line. The mean power spectrum was calculated by averaging the Fourier transforms of the analyzed regions. Using a reference phantom method, the average power spectrum was normalized to eliminate the effects of the system transfer function and transducer beam-forming^17^. The reference phantom consisted of glass beads with a diameter of 5–30 μm embedded in a homogenous background of microscopic oil droplets in gelatin (Medical Physics Department, University of Wisconsin, USA). The attenuation coefficient of the reference phantom was 0.576 dB/MHz.cm, and its speed of sound parameter was 1488 m/s. Within the − 6 dB bandwidth of the transducer, MBF, SS, and SI parameters were estimated using linear regression analysis as described in previous studies^18,19^. By fitting a spherical Gaussian form factor model to the estimated backscatter coefficient, ASD and AAC parameters were derived^20,21^.

In order to generate color-coded parametric maps for each QUS parameter, a sliding window technique was used. Each region of interest (ROI) consisting of tumor core and 5-mm margin^22^ was divided into square analysis blocks of size 10 ultrasonic wavelengths, with a 94% adjacent overlap in axial and lateral directions (2.2 mm × 2.2 mm). A previous study by Tadayyon^23^ et al. have demonstrated that QUS features in the core and margin of breast tumors can predict breast cancer response to neoadjuvant chemotherapy. Finally, QUS maps were cropped to eliminate the background and resized to 224 × 224 pixels using the MATLAB software package.

### Transfer learning

Transfer learning is a machine learning technique in which knowledge acquired from solving one task is applied to a different task^24^. In transfer learning, a model that has been trained on a large dataset for a specific task is used as a basis for training a new model on a different but related task. By leveraging the pre-trained model's learned features, the newly-trained model can benefit from the general knowledge captured during the pre-training phase, which can lead to enhanced performance, quicker convergence, and a reduced need for training data. The weights of the pre-trained model are typically kept constant^25^. This enables the model to learn task-specific characteristics while retaining the valuable information acquired during pre-training. Transfer learning is particularly advantageous when the new task has limited labeled data. Instead of training a model from the start, which requires a large amount of annotated data, transfer learning enables reusing the knowledge from a previously-trained model that was trained on a larger dataset or a different task. It is frequently employed in various machine learning domains such as computer vision, and natural language processing. Figure 4a shows a traditional deep learning image classification model where the convolutional (Conv) base is used to extract the features from the images and the fully-connected (FC) layers are used to perform the classification. The proposed transfer learning approach is shown in Fig. 4b. In this approach, features are engineered (detailed in the next section) after their extraction by the convolutional base. This is followed by the classification of LABC response using SVM^26^ algorithm instead of the FC layers. A custom Python™ program running on the Google Colab platform (Google, Inc., Mountain View, CA) was used for feature engineering, classification, and evaluation.

### Feature engineering

Features were extracted using the ResNet50V2 neural network^27,28^. ResNet50V2 is a convolutional neural network that has been trained on more than a million images from the ImageNet database^29^ and hence no training on the LABC dataset was performed. ResNet50V2 is a modified variant of ResNet50 that performs better on the ImageNet dataset than ResNet50 and ResNet101. In ResNet50V2, the propagation formulation of the connections between blocks was modified. The preprocessed input images of QUS maps are 224 × 224 pixels. ResNet50V2 generates a 7 × 7 × 2048 feature map on its last feature extractor layer from the input image which is then transformed to a 1 × 100,352 feature vector. All QUS slices across the tumor were used to extract feature vectors which were then averaged to give one feature vector for each tumor.

A larger feature space does not always imply a better model description, since some features may not be relevant. Nonsignificant and redundant features must be eliminated. This was accomplished by keeping features that contain 90% nonzero values. This was followed by selecting the 13 best features ($13=\sqrt{174} , 174$ being the number of patients) using the SelectKBest algorithm^30^. The SelectKBest is a feature selection technique commonly used in machine learning and data analysis. It is used to identify features based on the *k* features with the highest scores. Using ANOVA statistical analysis, the algorithm evaluates each feature independently and assigns a score to each feature. The score indicates the significance or relevance of the characteristic in relation to the objective variable.

The SMOTE technique^31^ was then employed to counteract the negative impact of the imbalanced dataset (in the dataset for this study, 37 NR vs. 137 RR) on classification performance. This technique generates random synthetic cases with attributes similar to those of actual cases but without replications, from the minority group. Consequently, it permits expanding the minority group's representation while preserving the actual data composition. To avoid incorporating synthetic examples that may inadvertently leak information into the testing process, data balancing was only performed on the training set^32^.

### Classification and evaluation

The SVM algorithm with the linear kernel was used in the classification of patients. It is a type of algorithm that uses a linear decision boundary to categorize data points. To ensure an objective evaluation of the classifier’s performance and determine how the model will generalize to an independent dataset, 20% of all cases were randomly selected for testing and treated as unseen data when selecting features (next step) to avoid bias. The remainder (subset not used for testing) underwent fivefold cross-validation. During fivefold cross-validation, the non-testing subset is randomly divided into five distinct subsets, each containing one-fifth of the entire dataset. The model is trained on four of the folds, with the remaining fold used for validation. Four performance metrics were used to evaluate the model on the unseen test set. These include precision, recall, F1-score, and balanced accuracy.

## Data Availability

Data collected and analyzed in this study are available from the Sunnybrook Research Institute Research Ethics Board approved study “Pilot Investigation of Ultrasound Imaging and Spectroscopy and Ultrasound Imaging of Vascular Blood Flow as Early Indicators of Locally Advanced Breast Cancer Response to Neoadjuvant Treatment”. Since this is patient data, the authors are legally bound to keep it confidential. Data can be made available upon request and review by the Institutional Review Board (IRB). Data requests may be sent to Dr. Kullervo Hynynen, Vice-president, Research & Innovation, Sunnybrook Research Institute (khynynen@sri.utoronto.ca).
